# Gut Microbiota, a Potential New Target for Chinese Herbal Medicines in Treating Diabetes Mellitus

**DOI:** 10.1155/2019/2634898

**Published:** 2019-02-18

**Authors:** Boxun Zhang, Rensong Yue, Yuan Chen, Maoyi Yang, Xiaoying Huang, Jiacheng Shui, Yuliang Peng, Jiawei Chin

**Affiliations:** Hospital of Chengdu University of Traditional Chinese Medicine, Chengdu 610072, China

## Abstract

The gut microbiota, as an important factor affecting host health, plays a significant role in the occurrence and development of diabetes mellitus (DM), and the mechanism may be related to excessive endotoxins, altered short-chain fatty acids (SCFAs), and disordered bile acid metabolism. Traditional Chinese medicine (TCM) has a long history of treating DM, but its mechanism is not very clear. Recent research has suggested that Chinese herbal medicine can improve glucose metabolism by remodeling the gut microbiota, which opens new avenues for further research on hypoglycemic mechanisms. This review presents the recent progress of Chinese herbs, herbal extracts, and herbal compound preparations in treating DM through regulating the gut microbiota and summarizes the main mechanisms involved, namely, anti-inflammatory and antioxidative effects, protecting the intestinal barrier and inhibiting lipotoxicity. In addition, some suggestions for improvement are also proposed.

## 1. Introduction

 Diabetes mellitus (DM) is a significant health concern that threatens people around the world. In 2017, the International Diabetes Federation (IDF) released the eighth edition of the “IDF diabetes map”, which showed that there were approximately 425 million adults (20-79 years old) worldwide suffering from DM, and this number might reach 629 million by 2045. In China, approximately 114.4 million people (20-79 years old) are living with DM; thus, China ranks the highest in the world (http://www.diabetesatlas.org). In the last few years, people have gradually recognized that the function of the human metabolism is not only determined by ourselves but is also closely related to our “second genome”— the gut microbiota, which has opened another door for a better understanding of DM [1–3]. To find better treatment effects for DM, a multitude of probiotic, prebiotic, and synbiotic products have been developed and gradually introduced in clinical practice [4].

Traditional Chinese medicine (TCM) has a longer than 2000-year history of treating DM. In recent years, accumulating evidence has confirmed that TCM can improve DM through different molecular mechanisms [5], and remodeling the gut microbiota is an important research focus [6]. In addition to the specific changes in some intestinal bacteria after herbal medicine is taken, researchers also pay attention to how the improved gut microbiota affects the metabolism of its host. In this review, we summarize the related literature, attempt to extract several main pathways linking the gut microbiota and glycometabolism, and then provide potential future directions that may improve the limitations of current research.

A literature search was performed using the PubMed, CNKI, Chinese Biomedical Literature, Wanfang, and VIP databases. The keywords for the search included “diabetes”, “gut microbiota”, “gut microflora”,“gut flora”, “intestinal microflora”, “Traditional Chinese medicine”, “herbal medicine”, and “herbs”, and we also searched related references.

## 2. The Relationship between DM and the Gut Microbiota

The gut microbiota not only affects the intestinal tract but also has a profound impact on the host's metabolic function through various mechanisms [1]. In recent decades, accumulating evidence has suggested a close connection between DM and the gut microbiota. Two cohort studies on individuals in China and Europe revealed that there are compositional and functional alterations in the gut microbiota of patients with type 2 diabetes mellitus (T2DM). For example, the amount of butyrate-producing bacteria* Roseburia* and* Faecalibacterium prausnitzii* was lower [7, 8]. In addition, a study based on the Han Chinese population showed that, compared to healthy subjects, people with type 1 diabetes mellitus (T1DM) had distinctly different gut microbiota, and* Bacteroides* abundance was positively correlated with autoantibodies [9].

How does a disturbed gut microbiota affect the carbohydrate metabolism of its host? According to current research, the reasons may be related to the following factors: (1) lipopolysaccharides (LPS): LPS are the vital component of the outer membrane of gram-negative bacteria, while they may cause low-grade inflammation associated with DM when excessive LPS enter into the blood circulation [10]; (2) short-chain fatty acids (SCFAs): SCFAs, mainly including acetic acid, propionic acid, and butyric acid, are the major fermentation products produced from indigestible fiber and polysaccharides, and reduced SCFA levels may destroy the host's metabolic homeostasis because they are related to energy metabolism, GLP-1 secretion, and the integrity of the intestinal mucosa [11]; (3) bile acids (BAs): BAs are produced by cholesterol degradation and are metabolized by the gut microbiota, and disordered metabolism of BAs may affect the expression of bile acid receptors in the intestine and then impair related glucose metabolism pathways [12]; (4) gut permeability: a disturbed gut microbiota may disrupt intestinal tight junction proteins, enhance gut permeability, and lead to the consistent leakage of LPS, which in turn triggers the systemic low-grade inflammation [13]; (5) energy harvest: an obesity-associated gut microbiota is more effective in utilizing energy from the diet, resulting in energy overload in the host, which forms a crucial foundation for the onset of insulin resistance and T2DM [14]; (6) intestinal immune: the gut microbiota is crucial to the development and modulation of the intestinal mucosal immune, and the dysfunctional interaction between the gut microbiota and the immune system is related to the occurrence of T1DM [15]. Conversely, improved gut microbiota may regulate one or more of the above pathways, exerting a comprehensive therapeutic effects on DM.

## 3. Evidence of Chinese Herbal Medicines Treating DM by Regulating the Gut Microbiota

### 3.1. Herbs and Herbal Extracts

*Folium Mori* for the treatment of* Xiaoke* (the name of DM in TCM) is recorded in the* Compendium of Materia Medica *[16], and its hypoglycemic effect has been confirmed in animal experiments [17] and clinical trials [18]. Feeding a diet containing 20%* Folium Mori* power to diabetic rats for 8 weeks can inhibit nonesterified fatty acid (NEFA) signaling pathway and ameliorate hyperglycemia. At the same time, the proportion of Bacteroidetes in intestinal bacteria is restored after* Folium Mori* treatment [19]. Another study confirmed that* Folium Mori* could promote the production of SCFAs and regulate the metabolism of steroids and BAs [20]. In addition to* Folium Mori*,* Dendrobium candidum *can alleviate oxidative stress in liver [21], and* Rhizoma Dioscoreae *is conducive to reducing the blood glucose of patients with T2DM [22]. Their details are summarized in Table 1.

Berberine is the principle bioactive alkaloid of some heat-clearing herbs, such as* Coptis chinensis *[23]. Recent studies have shown that berberine is an excellent gut microbiota modulator in animals and humans with DM. For example, it can promote the proliferation of* Bifidobacterium* and* Lactobacillus*, inhibit the growth of* Escherichia coli,* and reduce the levels of LPS in the intestine, thereby relieving chronic systemic inflammation [24]. In addition, the damaged intestinal mucosa and immune barrier can also be repaired by berberine, which contributes to preventing intestinal endotoxins from entering the blood and maintaining the host's metabolic homeostasis [25, 26]. In addition, many other phytochemicals, such as rhein [27],* Seabuckthorn* protein [28],* Zanthoxylum* alkylamides [29], and polyphenols from* Fructus MoriL*. [30], also have a hypoglycemic effect by regulating the gut microbiota (Table 1).

Besides, polysaccharides extracted from herbal medicines are also potential prebiotics.* Qixing Nie* and his colleagues [31] confirmed that the polysaccharides from the seeds of* Plantago asiatica* L. could reduce body weight, decrease blood glucose, and repair damaged kidney function in diabetic rats. In addition, it could also increase the number of bacteria such as* Bacteroides vulgatus*,* Lactobacillus fermentum*,* Prevotella loescheii*, and* Bacteroides ovatus* and promote the production of SCFAs. Moreover, the polysaccharides extracted from* Ganoderma atrum *[32],* Maydis stigma*[33],* Radix Pseudostellariae *[34],* Morus nigra* [35], and* Momordica charantia L*. fermented with* Lactobacillus plantarum NCU116 *[36] also can treat DM by regulating intestinal microecology (Table 1).

### 3.2. Herbal Compound Preparations

TCM formula is the combination of several herbs, and the compatibility of the herbs is the key to playing a synergistic therapeutic role [37]. Xiexin Tang, including three herbs:* Rhei rhizome*,* Scutellaria radix*, and* Coptidis rhizome*, shows ideal anti-inflammation, hypoglycemic and hypolipidemic effects in rats with T2DM. In addition, Xiexin Tang can increase the number of some anti-inflammatory bacteria such as* Adlercreutzia, Alloprevotella,* and the concentration of SCFAs in feces. The correlation analysis suggests that the increased intestinal bacteria may be a direct regulator of metabolic function [38]. In addition to animal experiments, the relevant evidences are also confirmed by clinical randomized controlled trials. The classic formula Gegen Qinlian Decoction and the modern herbal formula AMC both have significant hypoglycemic effects, and the gut microbiota structure is optimized after 12 weeks of administration. For example, levels of* Faecalibacterium*, the bacteria with anti-inflammatory effects, are profoundly increased by the two formulae; the SCFA-producing bacteria,* Roseburia* and* Blautia*, are also promoted after AMC treatment [39, 40]. Besides, the hypoglycemic effect of Gegen Qinlian Decoction (animal experiment)[41], Huanglian Jiedu Decoction [42, 43], Qijian mixture [44], Banxia Xiexin Decoction [45], and three innovative herbal compound preparations [46–48] also benefits the regulation of the gut microbiota, and their details are listed in Table 2.

### 3.3. Summary

According to the existing literature, the effects of antidiabetic herbal medicines on the gut microbiota have the following rules: (1) regulation of the microbiota structure by increasing microbial diversity and reducing the Firmicutes/Bacteroidetes(F/B)ratio; (2) increasing the anti-inflammatory bacteria such as* Bifidobacterium*,* Lactobacillus*,* Akkermansia,* and* Faecalibacterium*; (3) increasing the SCFAs producing bacteria, such as* Roseburia* and* Eubacterium*, and promoting the concentration of SCFAs in the intestine; (4) decreasing the abundance of pathogenic bacteria such as* Escherichia coli* and* Enterococcus*. These changes in the gut microbiota may trigger a series of chain reactions that may then improve the glucose metabolism of the hosts. The specific mechanisms are discussed below.

## 4. Mechanisms of Chinese Herbal Medicines Treating DM by Regulating the Gut Microbiota

### 4.1. Anti-Inflammatory Effect

Chronic low-grade inflammation has been recognized as a characteristic of some metabolic diseases such as DM and obesity, and the state of the gut microbiota is closely related to this type of inflammation [49].* Lactobacillus*, the probiotics widely used in various fields, has the function of reducing metabolism-related inflammation in both STZ-treated rats [50] and humans with DM [51].* Bifidobacterium* can also reduce the release of proinflammatory cytokines by restoring the balance between regulatory T cells (Tregs) and B lymphocytes and reversing the bacterial translocation process from the intestine to tissues [52, 53]. As we have introduced above, the Chinese herbal medicines berberine [24],* Seabuckthorn* protein [28],* Zanthoxylum* alkylamides [29],* Radix Pseudostellariae* polysaccharides [34], Gegen Qinlian Decoction [39, 41], and so on are all promoters of* Lactobacillus* and* Bifidobacterium* proliferation, which may be a significant reason that they exert better therapeutic benefits. In addition to promoting the growth of probiotics, alleviating the adverse effects of LPS can also attenuate inflammation response. Microbiota disorder may increase the LPS level in intestine and then aggravate the inflammatory injury of intestinal epithelium. When excessive LPS enter the blood through damaged intestinal mucosa, the low-grade systemic inflammation may be triggered [54]. In the above herbal medicines, Gegen Qinlian Decoction [41] and Banxia Xiexin Decoction [45] can reduce the leakage of LPS and decrease the inflammatory factors in serum; Huanglian Jiedu Decoction [42]can downregulate the expression of LPS-related inflammatory proteins and improve the intestinal mucosal barrier function.

Taken together, increasing anti-inflammatory bacteria, reducing the production and leakage of LPS, and downregulating the LPS-related inflammatory factors are the key links for the anti-inflammatory effects of TCM.

### 4.2. Antioxidative Effect

Oxidative stress plays an important role in the pathological process of DM, and it not only destroys islet *β* cells and insulin signaling pathways, but also may contribute to serious complications such as diabetic cardiovascular disease and diabetic nephropathy [55]. Recent studies show that some intestinal bacteria have antioxidant activity. For one thing, the probiotics reduce the level of intestinal oxidative stress through their own antioxidases and antioxidant metabolites; for another, they can also activate the antioxidant system of the host and improve oxidative stress via different pathways [56]. A healthy intestinal microbiota is the basis for maintaining redox homeostasis, while a disordered microbiota composition may induce gut epithelial reactive oxygen species (ROS) generation, causing intestinal injuries or even systemic diseases [57, 58]. In recent years, researchers have confirmed that some pro- and prebiotics can improve the oxidative stress state [59]. Similarly, the aforementioned hypoglycemic TCM such as* Dendrobium candidum *[21], Polyphenols from* Fructus Mori L*. [30], and several herbal polysaccharides [31, 32, 34–36] also have antioxidant activities. In the diabetic state, enterogenous endotoxin not only is a source of inflammation but may lead to oxidative stress in some organs, such as pancreas [60] and liver [61]. Through regulating the gut microbiota and repairing the intestinal mucosal barrier, herbal medicines can reduce endotoxin damage and avoid a series of problems caused by it. In addition, accumulating evidence indicates that some bacteria such as* Lactobacillus*,* Bifidobacterium*, and* Akkermansia* and the bacterial metabolite butyrate also have potential antioxidant features [56, 62–64]; as listed above, plenty of herbal medicines can promote their increase.

Thus, some herbal medicines, especially the herbal polysaccharides, may play an antioxidant role by promoting probiotic growth and inhibiting enterogenous endotoxin damage.

### 4.3. Protecting the Intestinal Barrier

In 1986, researchers already observed increased intestinal permeability in diabetic patients [65], and in recent years people have been concerned about the “second attack” to the organism caused by the damaged intestinal barrier. In the physiological state, the intestinal mucosa absorbs nutrients and prevents pathogenic bacteria or endotoxins from invading the blood. However, under the influence of a high fat diet, inflammatory stimulation, oxidative stress, and other factors, the structure and function of the intestinal mucosa could be disrupted, which may result in intestinal injury and systemic diseases such as DM [66]. Although the intestinal barrier plays an important role in health, there are only few drugs focusing on it [67]. By regulating the microbial composition and increasing SCFAs, some herbal medicines can improve intestinal barrier function and inhibit the damage of LPS. Specifically, adherent-invasive* Escherichia coli* may trigger inflammatory response and break the mucosa homeostasis [68]. On the contrary,* Akkermansia* and* Lactobacillus* can restore the integrity of intestinal epithelium by reducing proinflammatory cytokine [69, 70], and* Bifidobacterium* can maintain intestinal health by promoting the secretion of glucagon-like peptide-2 (GLP-2) [71]; SCFAs, as the fuel for the intestinal mucosa, not only provide energy for epithelial cells [72] but also facilitate tight junction assembly [73]. As mentioned above, berberine [24–26] can effectively regulate the gut microbiota, improve the intestinal permeability by inhibiting TLR4/MyD88/NF-kB signaling pathway, and increase the secretion of GLP-2. The compound preparation containing Burdock Fructooligosaccharide GF13 and* Lactobacillus plantarum* Sc 52 [47] can significantly promote SCFA production and restore the impaired intestinal barrier, which may be a potential prebiotic product for the treatment of DM.

In short, a damaged intestinal barrier is the hidden killers of metabolic homeostasis, and herbal medicines can improve this pathological state by decreasing pathogenic bacteria, increasing mucosal protective bacteria, and promoting the production of SCFAs.

### 4.4. Inhibiting Lipotoxicity

Lipotoxicity refers to cell dysfunction or death caused by excess lipid accumulation in nonadipose tissues [74], which not only further aggravates the progression of DM [75, 76] but also leads to diabetic complications such as cardiomyopathy [77] and angiopathy [78]. Recent studies suggest that the gut microbiota plays multiple fundamentally roles in maintaining lipid metabolic homeostasis and may be the potential target for improving dyslipidemia [79]. Above all, regulating the gut microbial ratio is an effective approach. In general, the obese usually have a higher Firmicutes/Bacteroidetes (F/B) ratio than the normal-weight [80, 81]. After treatment with rhein [27], polyphenols from* Fructus Mori L*. [30], and* Morus nigra* polysaccharides [35], a lower F/B ratio is observed. Besides, herbal medicines such as* Zanthoxylum* alkylamides [29], polysaccharides from the seeds of* Plantago asiatica* L. [31], and the fermented* Momordica charantia L*.[36] can inhibit lipotoxicity by promoting the production of the gut microbiota-derived metabolites acetate and butyrate. Specifically, acetate can regulate the expression of beige adipogenesis-related genes in white adipose tissue, thereby promoting the release of heat [82]. Butyrate is helpful for enhancing the mitochondrial function of skeletal muscle and brown fat and increasing energy expenditure [83]. In addition, in the abdominally obese individuals, serum lipopolysaccharide-binding protein (LBP) and NEFAs are highly correlated, which suggests LPS also lead to the disorder of lipid metabolism [84]. As discussed above, some herbal medicines can inhibit LPS by regulating the microbiota. Finally, the bacterial metabolites BAs can modulate systemic lipid metabolism via the nuclear farnesoid X receptor (FXR) and the G protein-coupled receptor 5 (GPR5) [85], and it may be a potential significant target for herbal medicines, while the relevant evidence is lacking and further study is needed.

Altogether, TCM attenuates lipotoxicity mainly through reducing F/B ratio, regulating microbial metabolites acetate, butyrate, and decreasing the damage of LPS.

### 4.5. Summary

**Figure 1:**
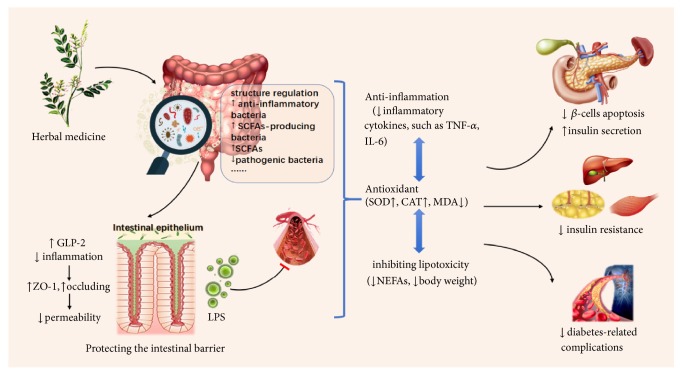
*The mechanisms of Chinese herbal medicines treating DM by regulating the gut microbiota*. SCFAs: short-chain fatty acids, LPS: lipopolysaccharide, GLP-2: glucagon-like peptide-2, TNF-*α*: tumor necrosis factor-*α*; IL-6: interleukins-6, SOD: superoxide dismutase; CAT: catalase, MDA: malondialdehyde; NEFAs: nonesterified fatty acids.

In summary, herbal medicines can exert multiple beneficial therapeutic effects on DM by regulating the gut microbiota, and anti-inflammatory and antioxidant effects, protecting the intestinal barrier and inhibiting lipotoxicity are the main mechanisms. Moreover, promoting GLP-1 secretion and inhibiting hepatic gluconeogenesis may also be related to the microbiota regulation. In addition to restoring islet function and improving insulin resistance, these mechanisms are also conducive to prevent the development of diabetes-related complications. In a word, the effects of herbal medicines on the gut microbiota are comprehensive and far-reaching (Figure 1).

## 5. Discussion and Future Perspectives

Data from numerous studies have supported an essential role for the gut microbiota in the process of TCM treating DM, but in the large number of bacterial species, which may exert the most important effects? It is necessary to pay attention to the core microbiota. Although* Lactobacillus* species account for only 0.01 to 0.06 of all bacterial species, it plays a significant role in protecting the epithelial barrier, producing antipathogenic compounds and regulating immunity [86]. Recently, accumulating data suggests probiotic* Lactobacillus* strains can play an auxiliary role in treating DM [87], while other studies show that the abundance of* Lactobacillus* is positively correlated with the level of blood glucose [8, 88], so the crosstalk between* Lactobacillus* and glucose metabolism requires further investigation. Bifidobacteria are among the dominant members of the breastfed infant's gastrointestinal tract [89], and selective increases of bifidobacteria could improve high-fat-diet-induced DM and metabolic endotoxaemia in mice [90].* Akkermansia muciniphila* is regarded as a next-generation benefical microbe that can activate Toll-like receptor 2 signaling pathways and promote the production of IL-10, thus playing a protective role for the intestinal mucosal barrier [91].* Roseburia* has a strong butyrate production capacity that can provide energy for intestinal epithelial cells and inhibit the release of proinflammatory cytokines [92]. Normally, the abundance of* Bifidobacterium*,* Akkermansia,* and* Roseburia* is negatively correlated with the level of blood glucose [91–93]. As shown above, most TCM can promote the growth of these beneficial bacteria, which then elicit multitarget therapeutic effects for the treatment of DM.

However, we must recognize that plenty of factors can affect the gut microbiota [94]. In order to make use of TCM better, it is necessary to discuss it in the real world, not just in the laboratory. Among the influencing factors of adult microbiota, dietary factors can account for up to 57% of gut microbiota changes [95], and recently the gut microbiota-targeted diet for treating DM has become a topic of widespread concern [96].* Liping Zhao* and his colleagues [97] found that a diet rich in fiber could optimize the gut microbiota, produce more SCFAs, and help to control blood glucose more effectively, which presented a novel ecological approach for managing T2DM. In addition, the influence of diet on microbiota may be related to the efficacy of herbal medicines. Intestinal bacteria can convert herbal chemicals into various bioactive substances, thus promoting better absorption and utilization [98], while disordered microbiota may affect the normal metabolism of drugs to some extent [99]. So, we speculate that the gut microbiota shaped by a healthy diet will enhance the efficacy of TCM, but the hypothesis warrants further investigation.

The further perspectives may include but are not limited to the following directions: above all, more advanced technical means such as metagenomic sequencing and germ-free animal should be applied to better reveal the relationship between TCM and the gut microbiota. Besides, the current studies mainly focus on intestinal bacteria, LPS and SCFAs, and the further exploration can aim at the intestinal fungi and other bacterial metabolites such as BAs. In addition, the research results based on mice/rat are difficult to apply in clinical practice directly, and the studies on human gut microbiota are urgently needed. Last and the most important, more attention should be shifted from mechanism to application. On the one hand, innovate and improve TCM in the direction of individualized targeted therapy; on the other hand, attempt to combine herbal medicines with the probiotics, probiotics or healthy food, and develop new and more effective compound preparations.

## 6. Conclusion

Chinese herbal medicines used to treat DM are closely related to regulating the gut microbiota, and the optimized gut microbiota improves the glucose metabolism of hosts, mainly by exerting anti-inflammatory and antioxidant effects, protecting the intestinal barrier, and inhibiting lipotoxicity. In the next step, more studies need to be based on the human microbiota and take individualization, precision, and multidisciplinary integration as the development goals. In short, the gut microbiota provides a new opportunity to elucidate the mechanism of TCM in treating DM; at the same time, herbal medicine is also a treasury of potential prebiotics and needs more in-depth study.

## Figures and Tables

**Table 1:** Chinese herbs and herbal extracts treating DM by regulating the gut microbiota.

Herbs/Herbal extracts	Models	Changes of the gut microbiota and their metabolites	Core mechanisms	Ref.
*Folium Mori*	SD rats	**Increased**: the phyla Bacteroidetes and Proteobacteria and class Clostridia	Improve NEFA metabolism	[19]

*Dendrobium candidum*	KM mice	**Increased**: the abundance and diversity of gut microbiota	Attenuate oxidative stress	[21]

*Rhizoma Dioscoreae*	human	**Increased**: *Bifidobacterium*	N/A	[22]

Berberine	Wistar/SD rats	**Increased**: *Bifidobacterium*, *Lactobacillus* **Decreased**: *Escherichia coli*, *Enterococcus*	Protect intestinal barrier; suppress inflammatory response; promote GLP-2 secretion	[24–26]

Rhein	db/db mice	**Increased**: Bacteroidetes, *Akkermansia* **Decreased**: Firmicutes, F/B ratio	Promote GLP-1 secretion	[27]

*Seabuckthorn* Protein	ICR mice	**Increased**: *Bifidobacterium*, * Lactobacillus*, *Bacteroides* **Decreased**: *Clostridium coccoides*, PH value	Improve fatty acids metabolism	[28]

*Zanthoxylum *alkylamides	SD rats	**Increased**: *Lactobacillus*, *Bifidobacterium*, *Clostridium*, SCFAs **Decreased**: *Enterococcus*, *Enterobacterium*, *Bacteroides*, PH value, free ammonia	Inhibit hepatic gluconeogenesis; promote insulin secretion	[29]

Polyphenols from *Fructus Mori L.*	db/db mice	**Increased**: Bacteroidetes, Anaeroplasmatales, butyrate, propionate **Decreased**: Firmicutes, F/B ratio, *Bacillus*, *Lactobacillus*	Attenuate oxidative stress	[30]

Polysaccharide from the seed of *Plantago asiatica *L.	Wistar rats	**Increased**: *Bacteroides*, *Lactobacillus*, *Prevotella*, SCFAs **Decreased**: *Alistipes obesi*	Improve NEFA metabolism; attenuate oxidative stress	[31]

*Ganoderma atrum *Polysaccharide	Wistar rats	**Increased**: SCFAs	Attenuate oxidative stress	[32]

*Maydis stigma *Polysaccharide	KM mice	**Increased**: *Lactobacillus*, *Bacteroides*	N/A	[33]

*Radix Pseudostellariae *Polysaccharide	C57 mice	**Increased**: *Lactobacillus*, *Bifidobacterium*	Attenuate oxidative stress; suppress inflammatory response	[34]

*Morus nigra* Polysaccharide	db/db mice	**Increased**: Bacteroidales, *Bacteroides Lactobacillus*,* Allobaculum*, *Akkermansia* **Decreased**: F/B ratio, opportunistic pathogens such as *Staphylococcus *and *Enterococcus *	Regulate lipid metabolism; attenuate oxidative stress	[35]

Polysaccharide from the fermented *Momordica charantia L*	Wistar rats	**Increased**: *Lactococcus laudensis, Prevotella loescheii, *SCFAs **Decreased**: pH value	Attenuate oxidative stress	[36]

*Abbreviations*. NEFA: nonesterified fatty acid; F/B: Firmicutes/Bacteroidetes; GLP-2: glucagon-like peptide-2; GLP-1: glucagon-like peptide-1; SCFAs: short chain fatty acids; N/A: not applicable.

**Table 2:** Herbal compound preparations treating DM by regulating the gut microbiota.

Preparations	Composition of preparations	Models	Changes of the gut microbiota and metabolites	Core mechanisms	Ref.
Xiexin Tang	Rhizome Rhei, Radix Scutellaria, Rhizome Coptidis	SD rats	**Increased**: some SCFAs producing and anti-inflammatory bacteria such as* Adlercreutzia* and *Alloprevotella *; SCFAs	Suppress inflammatory response	[38]

Gegen Qinlian Decoction	Radix Puerariae, Radix Scutellariae, Rhizoma Coptidis, Honey-fried Licorice Root	human	**Increased**: *Faecalibacterium*, *Bifidobacterium*, *Gemmiger* **Decreased**: *Alistipes*,* Odoribacter*	N/A	[39]
		KK-Ay mice	**Increased**: *Lactobacillus johnsonii*, *Bacteroides vulgatus*	Suppress inflammatory response	[41]

AMC	Rhizoma Anemarrhenae, Momordica charantia, Rhizoma Coptidis, Salvia miltiorrhiza, Red yeast rice, Aloe vera, Schisandra chinensis, Rhizoma zingiberis	human	**Increased**: *Faecalibacterium*, *Roseburia*, *Gemmiger*, *Coprococcus*, *Megamonas*, *Blautia*	N/A	[40]

Huanglian Jiedu Decoction	Coptidis Rhizoma, Scutellariae Radix, Phellodendri Cortex, Gardeniae Fructus	ZDF rats	**Increased**: *Bacteroides*,* Clostridium*, *Roseburia*,* Akkermansia*,* Oscillospira*, *Aggregatibacter*, *Eubacterium* **Decreased**: *Prevotella*	Protect intestinal barrier; suppress inflammatory response; promote GLP-1and GLP-2 secretion	[42]
		SD rats	**Increased**: *Parabacteroides*, *Blautia*, *Akkermansia* **Decreased**: *Aerococcus*, *Staphylococcus*, *Corynebacterium*	Regulate lipid metabolism; suppress inflammatory response; attenuate oxidative stress	[43]

Qijian mixture	Astragalus membranaceus, Ramulus euonymi, Coptis chinensis, Pueraria lobata	KKay mice	**Increased**: Bacteroidetes	Improve carbohydrate and amino acid metabolism disorder	[44]

Banxia Xiexin Decoction	Rhizoma Pinelliae Praeparata, Radix Scutellaria, Rhizome Coptidis, Panax ginseng, Rhizoma zingiberis, Radix liquiritiae, Fructus Ziziphi Jujubae	SD rats	**Increased**: *Bacteroides*,* Bifidobacterium*,* Lactobacilli*,* Enterococcus*	Suppress inflammatory response	[45]

Herbal extract + Chemical drugs	Houttuynia cordata Thunb., Metformin	OLETF rats	**Increased**: *Roseburia*, *Akkermansia*, SCFAs **Decreased**: Gram-negative bacteria, *prevotella*, *Escherichia coli*, LPS	Suppress inflammatory response	[46]

Herbal extract +Probiotics	Burdock Fructooligosaccharide GF13 *Lactobacillus plantarum* Sc 52	C57 mice	**Increased**: *Lactobacillus*, *Bifidobacterium*, SCFAs **Decreased**: *Enterobacterium*, *Enterococcus*	Protect intestinal barrier; suppress inflammatory response;	[47]

Herbal extract +Prebiotics	Berberine, stachyose	KKay mice	**Increased**: *Lactobacillus*,* Bifidobacterium*	N/A	[48]

*Abbreviations*. SCFAs: short chain fatty acids; GLP-1: glucagon-like peptide-1; GLP-2: glucagon-like peptide-2; LPS: lipopolysaccharides; N/A: not applicable.
